# Are Your Vitals OK? Revitalizing Vitality of Nurses through Relational Caring for Patients

**DOI:** 10.3390/healthcare9010046

**Published:** 2021-01-05

**Authors:** Jung Hwan Park, Young Kyun Chang, Sooyeol Kim

**Affiliations:** 1 Sogang Business School, Sogang University, 35 Baekbeom-ro, Mapo-gu, Seoul 04107, Korea; pjhwan@sogang.ac.kr; 2 Department of Management and Organization, National University of Singapore Business School, National University of Singapore, 15 Kent Ridge Drive, Singapore 119245, Singapore; sooyeolkim@gmail.com

**Keywords:** healthcare employees, relational caring, vitality, moral identification

## Abstract

This study offers an alternative approach to address on-going concerns about burnout of healthcare employees. Departing from the existing job-demand based approach proposing that healthcare employees’ burnout can be resolved by reducing demands, we theorize that patient-centered prosocial behavior, even if it often increases job demands, could serve as potential job resources that fuel positive energy to vitalize nurses at work. We further theorize that this possibility could be more pronounced among a group of nurses with a strong sense of ethical membership regarding their hospital (i.e., moral identification). To test our hypotheses, we used a sample of 202 nurses from 104 South Korean hospitals. We found that, even controlling for workloads as an indicator of job demand, nurses who engage in patient-centered prosocial behavior (i.e., relational caring) are likely to feel vitalized, and this pattern is more salient among a group of nurses with high moral identification. Results indicate that prosocial behavior could be an alternative job resource that helps nurses flourish at work.

## 1. Introduction

As of 2020, approximately 28 million nurses across the globe are working for our health and well-being [1]. Nurses, as one of the largest healthcare professions, serve the backbone of each country’s healthcare system by providing medical care for sick individuals as well as keeping health industries going. Because of their significant role in society and high work demands, it has been widely recognized that nurses are experiencing burnout and severe emotional exhaustion. This stems largely from understaffing, heavy workloads, stressful tasks, and having to engage in emotionally draining labor with difficult patients [2]. For this reason, hospitals have been suffering from high turnover rates of nurses and medical staff. For instance, one longitudinal survey indicates that approximately 27% of newly licensed registered nurses in the U.S. tend to leave their hospitals within the first year [3]. Similar trends are also witnessed in other regions of the world (see [4]). The recent pandemic crisis has made things even worse. If such trends continue, medical care would become more costly and societal burdens continue to rise. As such, it is important to find proper solutions to address nurses’ work stress and burnout in the hospital.

Regarding concerns for nurses’ well-being and the quality of their work, previous studies have made a conclusion that the job demands of nurses should be reduced in order to facilitate their well-being and performance [5]. This view is widely upheld by Job Demands-Resources (JDR) theory, which explains why employees experience burnout and disengagement [6,7]. According to JDR theory, employees are more likely to experience burnout when job demands are high, and job resources buffer this relationship. Job demands include physical, psychological, social, and organizational features of a job that require continuous physical and psychological effort, whereas job resources include job control, potential for qualification, participation, and task variety, or social support from within and outside the organization. The JDR theory provides a useful insight and practical implications regarding how organizations should manage an employee’s well-being in the job context.

Our study adds to the existing body of literature on JDR theory by taking a possible alternative approach. The existing JDR-based work relies on an employee’s self-centered view. This implies that the best way to deal with employee burnout is to make employees themselves feel better by reducing the amount of work. However, a growing body of literature on positive organizational scholarship (POS) takes an alternative route to explain how and why employees flourish and thrive at work [8]. This body of literature claims that employees often find a meaningful path for their well-being by helping others rather than focusing on themselves [9]. Specifically, the prosocial model of job design [10] offers a more relevant rationale to support this claim. Grant suggested that when job holders have a chance to engage in prosocial behaviors for others, they are likely to experience positive states (e.g., competence, social worth, and self-determination), which are expected to prevent job holders from being exhausted [10]. Thus, “other-centered” (i.e., allocentric) prosocial behavior could be a potential job resource that helps employees thrive at work.

Building from a broader argument about POS, as well as a specific insight from the model of relational job design, our approach extends the boundary of theorizing about job design from the job holder’s self-centered view to an other-centered view. In this paper, we focus on relational caring as a nurse’s patient-centered prosocial behavior, and vitality as an indicator of a nurse’s psychological well-being in the hospital. We propose that, as nurses engage in patient-centered prosocial behavior, they are likely to feel vitalized at work. Further, we also explore the moderating role of a nurse’s beliefs about ethical membership (i.e., moral identification; [11]) relating to the hospital. Given the ethical nature of other-oriented prosocial behavior [12], nurses who show a deep relational care for patients are likely to experience vitality more strongly, as long as they believe that their hospitals are concerned about ethics. Thus, prosocial behaviors could be a powerful job resource for employees particularly when the organization appreciates such behaviors.

This study contributes to the current literature in theory and practice. From a theoretical standpoint, this study builds on the emerging trend of positive organizational scholarship so as to extend the boundary of JDR theory. It argues that other-centered prosocial behavior could serve a potential job resource that causes exhausted employees to be more energetic at work. In other words, as far as nurses regard patients’ lives and well-being more truly and deeply, they are fueled by positive feelings. Further, this study explores the crucial role of an organizations’ ethical features that may lead employees to define themselves and regulate their evaluation of prosocial behaviors. Thus, good deeds compensate employees when their organizations treat these behaviors as valuable. From the practical standpoint, this study offers a feasible implication regarding how hospitals help nurses reduce stress and flourish. Hospitals need to empower nurses to be patient-focused at the point of service, and send a clear signal that hospitals value ethicality.

## 2. Theory and Hypothesis Development

### 2.1. Job Demands-Resources (JDR) Theory

The JDR theory analyzes why employees come to experience burnout and well-being by integrating various work-related factors in a single framework [6,7]. The JDR theory proposes that job demands operate as a stressor often resulting in strain (e.g., burnout) because employees are supposed to exert high effort in meeting their job-related demands. The theory also posits that the costs of job demands do not always lead to high strain when employees’ resources are provided on the job. These resources include various aspects in the workplace (e.g., supportive leadership) that can buffer the negative effects of job demands [6]. In other words, workplace resources can regulate the detrimental effects of job demands on strain.

On the one hand, job demands include physical, psychological, social, and organizational features of a job that require employees to accomplish their jobs [6,13,14]. Job resources are thought to be responsible for employee burnout [7] leading to a lack of vitality and detachment from work [15]. High work pressure, unpleasant physical or psychological environment, and emotionally demanding interrelation with clients are examples of job demands [6,7,16]. In the case of nurses, excessive workload or handling difficult patients could be possibly heavy job demands, since such jobs can increase a risk of making an error [17] and physical and psychological fatigue [18].

On the other hand, prior studies have found that job resources help individuals overcome stressful situations, feel motivated in their tasks, and eventually become fully engaged with their jobs [13,19]. Job resources help employees to be positive at work, since they are functional in the achievement of work goals, effective in reducing job demands, and helpful for facilitating individual growth [6,16]. As a result, proper job resources can result in positive outcomes at the individual and organizational levels [6], such as promoting vitality [20] and work engagement [21], or lowering turnover and absenteeism [22].

### 2.2. Relational Caring and Vitality

The JDR theory provides a reasonable explanation of how employees respond to job and organizational characteristics as they focus their attention on themselves as a job holder. However, a growing body of literature on organizational behavior has taken an alternative route to explain how employees respond, by shifting an individual’s focus toward others. Thus, departing from the assumption that employees are self-centered, scholars have begun to propose that employees can also become other-centered, and this leads them to experience positivity in the workplace.

We argue that helping others is likely to lead to positive outcomes for a number of reasons. First, helping others provides an opportunity to observe how individuals can change their social environments. Given that helping behaviors alleviate others’ concerns or problems, altruistic behavior provides concrete examples that lead to realization or recognition that individuals’ efforts are truly making an impact on someone’s life [23]. Second, helping can develop individuals’ positive assessments of their influence, because they take initiatives and use their volition to drive change. According to self-determination theory, individuals feel greater attachment to the activities they voluntarily choose to engage in and place greater meanings on them [24,25]. Third, those who engage in helping others are likely to attribute to the cause of successful experiences of the self. Indeed, studies suggest that individuals tend to overestimate one’s degree of influence over external environments (i.e., illusion of control; [26]) and claim more responsibility for successes than failures (i.e., self-serving bias; [27]). Taken together, helping is likely to facilitate an individual’s positive outcomes.

Organizational citizenship behavior (OCB) is a classic example. Preliminary studies defined OCB as a form of other-centered prosocial behavior triggered by some altruistic motivation to help others [8,9,28]. Scholars have examined how OCB reduces employee negativity and promotes employee positivity. For example, employees would be less likely to experience negative affectivity and burnout when they are involved with OCB [29]. Similarly, OCB increases an individual’s positive energy, which results in better employee well-being through the strengthening of personal resources [30].

In the hospital context, relational caring for patients could be a sort of other-focused prosocial behavior, like OCB. The caring behavior of nurses is altruistic behavior that helps patients to relieve stress and promotes a sense of safety [31,32]. Previous works view relational caring as helping behavior that goes beyond the prescribed role of nurses such as operating medical equipment and giving medical treatments [33,34]. As such, it is evident that nurses who are involved in relational caring for patients seem to help others (i.e., patients) rather than themselves, and further experience positive outcomes.

We assume that a nurse’s other-focused behavior, the relational caring for the patient, can lower burnout and promote positivity at work. A particular group of researchers on organizational behavior—the positive organizational scholarship (POS) group—offers more relevant rationales to support our claim in the service context. Grant described “other-focused” psychological states that employees are thought to experience as a result of performing prosocial jobs. Perceived impact on others (i.e., beneficiaries) is the degree to which employees are likely to experience their actions as positively affecting other people’s lives, and affective commitment to others is the degree of employees’ emotional attachments to them [10]. Later, Grant linked this other-focused psychology to employee positive states, proposing a theoretical model of relational job design and suggesting that, when employees have a chance to engage in prosocial behaviors for others, they are likely to experience positive states (e.g., competence, social worth, and self-determination), which can prevent them from being exhausted [10,35]. For instance, physicians and fire fighters may experience positivity at work (e.g., competence and social worth), because their tasks truly focus on others’ life and well-being.

As far as nurses engage in relational caring for patients, they are likely to contact patients more frequently and have an impact on patients more deeply. Then such prosocial nurses are likely to experience a strong states of positivity, such as self-determination, competence, and social worth [10,35], which can feed a nurse’ energetic states. As such, we predict that relational caring can promote nurses’ vitality at work.

**Hypothesis 1 (H1)**. *Relational caring behavior of nurses is positively associated with vitality at work.*

### 2.3. A Moderating Role of Moral Identification

As proposed above, as nurses regard patients’ lives and well-being more truly and deeply, they are likely to feel vitalized and engaged at work. We further propose that this possibility can be better pronounced in a context where nurses believe their hospitals take ethics seriously. May et al. introduced the concept of moral identification, defined as the “perception of oneness or belongingness associated with an organization that exhibits ethical traits (e.g., care, kindness, compassion), which also involves a deliberate concern of the membership with an ethical organization.” [11] (p. 682). A notion of moral identification implies that organizational morality is often an important attribute and characteristic that employees use to define, perceive, and evaluate their organizations [36]. As such, nurses who define themselves as a member of an “ethical organization” are likely to see prosocial behavior as a desirable and valuable action. Thus, if nurses have a strong sense of moral identification, their patient-centered prosocial behaviors strengthens the consistency between their behaviors and the values and attributes of their identification domain [37,38,39,40]. This might lead them to feel good about themselves and their hospital, which better feeds into the positive state of vitality at work. However, if nurses have weak to no sense of ethical membership, their patient-centered prosocial behaviors are irrelevant to strengthening their self-consistency. Hence, we predict that moral identification can moderate the relationship between a nurse’s relational caring behavior and vitality at work.

**Hypothesis 2a (H2a)**. *Moral identification moderates the relationship between a nurse’s relational caring behavior and vitality at work.*

We hypothesize that nurses’ relational caring behavior can feed their vitality at work, and this tendency would become much stronger when nurses believe their hospital consider ethics seriously. This proposition clearly indicates that organizational ethicality can function as an important contextual cue that employees use to judge whether their behavior is the “right” thing to do in their organization. This argument is not new. A number of previous works have repeatedly found that contexts or external features regulate employees’ ethical/prosocial behavior in the organization [41,42].

However, several scholars in the field of behavioral ethics have investigated the nullifying effect of contexts. For instance, people who define themselves as a moral person are willing to engage in prosocial behavior, but their level of engagement is not affected by external forces, especially when their personal morality is too strong. Notably, some exemplary works have already found that providing monetary rewards with people engaging in voluntary work for society actually undermines the willingness of people to engage in that task [43,44]. Brekke et al. interpreted that providing incentives for highly prosocial individuals when acting upon external demands could harm their pure motive for pursuing good deeds [45]. This finding can inform that organizational ethicality is not always an factor encouraging employees to engage in prosocial behavior, especially if their level of pro-socialness is high.

Relating to our study, we assume that nurses who engage in prosocial behavior (i.e., relational caring for patients) are likely to experience a positive state at work (thriving) when nurses believe that their hospital cares for ethicality. However, we further predict that such tendency will be weakened or even disappear in a condition where nurses’ workloads are excessive. In general, it is hard to imagine that nurses with heavy workloads would go the extra-mile for their patients. If they do so, it is not usual. It is probably because they truly believe such prosocial behavior is the right thing to do. Thus, nurses who provide deep care for patients, despite their heavy workloads, are motivated by their intrinsic prosocial volition and mind, not by an ethical feature of their organization. Thus, moral identification will not be a significant context that regulates a nurses’ prosocial behavior and its subsequent positive outcome. As such, a nurse’s sense of ethical membership would moderate the relationship between a nurse’s relational caring behavior and vitality only in a condition where a nurse’s workloads are normal, but not do so any more in a condition where a nurse’s workloads are excessive. Hence, we hypothesize as follows.

**Hypothesis 2b (H2b)**. *Moral identification moderates the relationship between a nurse’s relational caring behavior and vitality at work only when a nurse’s workloads are normal.*

## 3. Method

### 3.1. Sample

The survey was conducted among 669 nurses who are currently working at hospitals with more than 100 beds in South Korea. All the questionnaires were answered voluntarily and confidentially. Out of 669 questionnaires distributed, 231 responses were returned (response rate: 34.5%), but only 202 responses (*n* = 202) were used for our analysis upon the completeness of response. The majority of nurses were female (98.51%), and most of them were between 30 and 40 (20s = 18.3%, 30s = 37.6%, 40s = 32.2%, 50s above = 11.9%). In terms of position in the workplace, above half of the nurses were staff nurses (staff/field nurse = 65.8%, head nurse = 18.8%, team manager = 12.9%, director = 1.5%).

### 3.2. Measurement

All variables used are adapted from original questionnaires from previous works. Questionnaires were translated using the back-translation method [46]. English-Korean bilingual professionals who have a doctoral degree in management in the United States engaged in translating questionnaires from English to Korean. All variables are measured based on a 7-point Likert scale, from 1 being strongly disagree to 7 being strongly agree.

Independent variable. This study used seven items for *relational caring behavior*, partially adapted from the Caring Nurse-Patient Interaction short scale (CNPI-short scale). The CNPI-short scale was invented by Cossette et al. and verified with confirmatory factor analysis [34,47]. Sample items are: “I help patients to look for a certain equilibrium and balance in their lives”; “I help patients to explore what is important in their lives”; “I help patients to clarify which things they would like significant persons to bring them” (α = 0.96).

Dependent variable. *Vitality* was measured using eight items that were introduced by Atwater and Carmeli [48]. These items are based on the broader definition of thriving, which is a positive psychological state of learning and vitality [49]. Sample items of vitality are “I feel active and energetic at work”; “I feel a lot of excitement when I am doing my work”; “The work in the organization (hospital) gives me positive energy” (α = 0.95).

Moderating variable. To measure *moral identification*, we fully adopted the original five items developed by May et al. [11]. Sample items are “Being a member of the hospital whose members have an ethical characteristic is an important part of who I am”; “I strongly desire to be a member of the hospital whose members have an ethical characteristic”; “When thinking of the hospitals to which others belong, I would be proud of my affiliations with the hospital whose members have an ethical characteristic” (α = 0.92).

Control variables. We controlled for several variables to avoid alternative explanations for the relationships under study. Given that workload has a negative impact on the vitality of nurses [50,51,52], we controlled for the workload of nurses by measuring weekly working hours [53]. We also controlled for workload by measuring the relative bed-use rate of hospitals compared to other similar hospitals in size [54]. This item is based on a 7-point Likert scale by asking HR staff as follows. “Our hospital’s bed use rate of recent three years is higher than other hospitals of similar size.” Other personal characteristic variables, such as age, education-level, and position-level of nurses that may affect an employee’s job attitudes and behaviors [55], including vitality [21,56], were also controlled. The age of nurses was coded into five-point scale (1 = 20s, 2 = 30s, 3 = 40s, 4 = 50s and above), and the education-level was classified into three groups (1 = Associate, 2 = Bachelor’s degree, 3 = Master’s degree and above). Position-level was measured through four levels based upon the reporting structure of hospitals (1 = staff/field nurse, 2 = head nurse, 3 = team manager, 4 = director).

### 3.3. Analysis

We tested our hypotheses by using a regression analysis and bootstrapping method [57]. Bootstrapping method has a non-parametric advantage and does not violate a normality assumption. As such, it can be used in a relatively smaller sample size [58]. We used the Macro PROCESS program with mean-centered variables to test the main and moderation effect [59]. Hypotheses were tested simultaneously with 5000 bootstrap samples, and the results were considered significant if the 95% confidence interval did not include zero [60]. To be specific, the present study tested the main relationship between relational caring behavior and vitality (H1), and the moderation effect of moral identification (H2a, and H2b), while controlling for work hour, bed-use, age, education, and position of nurses.

## 4. Results

Table 1 shows descriptive statistics and correlations for all variables. As predicted, relational caring behavior and vitality is positively correlated (r = 0.53, *p* < 0.001). However, nurses’ workloads (work hour and bed-use) are not correlated with vitality.

### 4.1. Measurement Model Test

We first conducted a factor analysis to confirm the discriminant validity of the measurement model. We evaluated model fit indices, such as a comparative fit index (CFI), Tucker-Lewis index (TLI), standardized root mean square residuals (SRMR), and a root-mean square error of approximation (RMSEA), based on the criteria suggested [61,62]. The three-factor measurement model satisfied the criteria (CFI = 0.959, TLI = 0.951, SRMR = 0.055, and RMSEA = 0.077). Then, we compared model fit indices of a three-factor model to three two-factor models and a single-factor model. As shown in Table 2, the three-factor model shows a better model fit over the single-factor model with an evidence that all chi-square changes are significant (*p* < 0.001). Thus, we concluded that three focal variables (relational caring, moral identification, and vitality) show discriminant validity.

### 4.2. Regression Analysis

All hypotheses were tested using a hierarchical multiple linear regression analysis. For step 1, only control variables were entered. For steps 2 and 3, predicting variables (IV and MV) and their interaction term was tested consecutively. Specifically, we regressed vitality on age, education, position, work hour, and bed-use in the first step. Next, we regressed vitality on relational caring and moral identification in the second step. Last, we introduced the interaction term of caring and moral identification in the third step.

Hypothesis 1 tests if relational caring behavior of nurses is positively associated with vitality at work. Table 3 shows that relational caring behavior is positively related to vitality (β = 0.25, *p* < 0.01, LLCI = 0.12, ULCI = 0.39). Therefore, hypothesis 1 is supported.

Hypothesis 2a tests the moderating effect of moral identification. The moderating variable and the interaction term have a significant positive relationship with vitality. Specifically, moral identification is positively related with vitality (β = 0.51, *p* < 0.01, LLCI = 0.3764, ULCI = 0.6467) and the interaction term also has a significant positive association with vitality (β = 0.15, *p* < 0.05, LLCI = 0.03, ULCI = 0.27). Therefore, Hypothesis 2a was supported.

**Figure 1:**
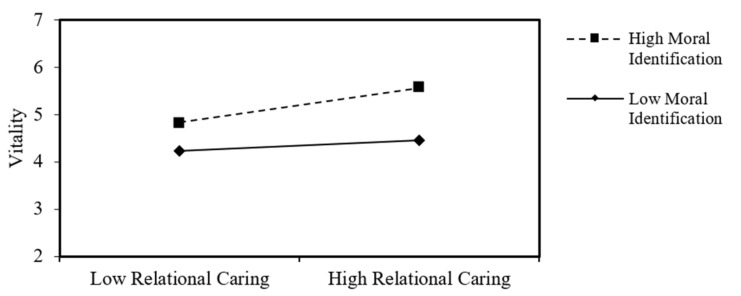
The moderation effect of a moral identification.

Figure 1 shows the interaction effect, which indicates that nurses with high moral identification (mean + 1 S.D.) may feel vitality more intensively as relational caring behavior increases from low (mean − 1 S.D.) to high (mean + 1 S.D.), compared to nurses who have a low level of moral identification (mean − 1 S.D.).

Hypothesis 2b tests if moral identification moderates the relationship between a nurse’s relational caring behavior and vitality only in a condition where a nurse’s workloads are normal rather than excessive. In order to discern two workload conditions, we test if the moderating effect will be present when nurses work 40 h and below a week, and whether the effect will be weaker or even disappear when nurses work 40 h above a week. As predicted, the moderating effect of moral identification is only found in the condition of normal workloads (β = 0.21, *p* < 0.01, LLCI = 0.08, ULCI = 0.34) (see Table 4), but disappears in the condition of excessive workloads (β = 0.07, *p* > 0.05, LLCI = −0.18, ULCI = 0.37) (see Table 5). Therefore, Hypothesis 2b is also supported.

## 5. Discussion

### 5.1. Summary and Theoretical Implication

Employees’ work demands have been thought to cause stress and burnout that are detrimental for well-being and work outcomes [63]. By employing positive organizational scholarship, our study alternatively suggested that employees’ others-focused behavior would benefit their well-being. Specifically, we drew on the sample of nurses, and found that nurses’ other-focused demands (i.e., caring behavior) would be beneficial for their vitality at work. This indicates that nurses who are willing to help others and show more caring behaviors to their patients tend to experience higher level of vitality. Moreover, our study revealed that nurses’ moral identification strengthens the relationship between caring behaviors and vitality. Thus, nurses who value the membership of an ethical organization tend to experience more positivity from helping patients. However, our study also proposes some interesting boundary conditions for the moderating effect of moral identification. We found the moderating effect to be present only when nurses’ workloads are normal, and the moderating effect was erased when nurses’ workloads are excessive.

This study offers meaningful contributions to the literature. First, our study shows that employees’ other-focused behaviors (e.g., helping or caring) have unique implications for employees’ positivity at work. Above and beyond the benefit of helping behaviors (e.g., OCB), our findings shed light on the benefits of nurses’ voluntary caring behaviors on their own vitality. This provides a new insight for work demands and JDR literature by suggesting employees’ prosocial and meaningful demands at work could be beneficial for their well-being. Further, our study also suggests an important implication to healthcare profession research regarding the way to deal with healthcare workers’ work stress and burnout.

Second, in addition to the main relationship between caring behavior and vitality, we tested the interaction effect of prosocial behavior and ethical membership on employee positive states. We assume that employees’ prosocial behaviors are not equally recognized by every organization. Some organizations recognize and encourage employees’ extra role-like prosocial behaviors, while others appreciate more employees’ in-role tasks. If this is the case, employees will become more or less vitalized due to their prosocial behaviors, depending upon how much they believe their organizations appreciate these behaviors. In this study, we predicted and found that employees’ prosocial behaviors and their concerns for ethical membership jointly affect their sense of workplace vitality. This attempt implies that prosocial behaviors and ethical cognition need to be integrated to better predict employee positive outcomes. In fact, a few previous works have already tried to put an individual’s prosocial behavior and moral cognition into one equation (e.g., [64,65]), but it is still far from conclusive in predicting how people behave when prosocial motivation interacts with ethical concerns.

Third, our study offers more nuanced findings for the moderation effect of moral identification. We predicted that nurses who engage in prosocial behavior are likely to feel vitalized at work, and this possibility will be more salient when nurses recognize the signal that their hospital considers ethicality to be important. This prediction turned out to be true. However, we further found that such a tendency disappears when nurses’ workloads are excessive. This means that, despite their heavy workloads, nurses who decide to provide deep care for patients are not necessarily motivated by their organizational norms and pressures, but rather by their intrinsic prosocial volition and mind. Our study contributes to the behavioral ethics literature by circumscribing the role of moral identification in the context of prosocial behavior, yet still offering more finely-tuned theorization.

### 5.2. Practical Implications

Our research has significant implications for practitioners. First, as mentioned, hospitals have reported high turnover rates of nurses and medical staff, mainly because healthcare employees have been suffering from excessive job demands, stress and burnout. This study proposes one remedy. We found that, as nurses engage in patient-centered caring, they are likely to thrive at work. As such, we suggest HR managers or employers be aware of the benefits of caring behaviors in the healthcare setting. One way of doing this is that managers could provide seminars or training sessions that highlight the benefit of caring behaviors and the types of those behaviors. Hence, healthcare workers could acknowledge the benefits of caring behaviors and show motivating other-focused behaviors at work.

Organizations could directly provide resources and tools that aid workers’ caring behavior. Given that caring behavior is integral in supporting patients under hardship, understanding potential drivers of healthcare workers’ engagement in caring behavior has significant implications for policymakers and managers. Further, managers could reinforce the caring-friendly culture in the hospital. For example, organizations could launch a campaign of recognition and support for workers who show exceptional patient caring (e.g., best monthly care-giver). Thus, in order to promote employee caring behaviors, it is important to provide opportunities for employees to realize how their contributions make positive differences in others’ lives.

Finally, hospital managers can also be informed by the finding that caring behavior can serve as a job resource that energizes tired nurses when they perceive their hospitals to be highly attentive to ethics. Ethics is an important organizational context by which employees make sense of their prosocial behaviors. As such, hospital managers should try to keep their organizational image ethical, so that healthcare employees feel safe and comfortable when they engage in other-focused behaviors.

### 5.3. Limitations and Future Studies

As our study has some limitations, we discuss future research avenues that might extend the findings. First, our study relied on self-report measurements, with cross-sectional design measuring all variables at the same time. We attempted to preemptively address the potential concerns of common-methods variance bias (CMV; [66]). For example, as Podsakoff et al. argued that CMV is less concerned when the study has moderation effects because these moderation effects cannot be artifacts of CMV, as it decreases the sensitivity to interaction tests [66,67,68]. However, we encourage future studies to reduce CMV through objective assessments or multisource assessments of outcomes. Further, study in a laboratory setting could help in establishing causality. In addition, future study may want to explore the within-person fluctuations in caring behaviors and their effects on outcomes by employing the experienced sampling method (ESM).

Second, all of our participants were from South Korea, which reduced the generalizability of the findings to some degree. Future scholars could examine cultural differences when replicating this study by working with samples from different countries. Considering that other-focused behaviors and organizational perspectives on these helping behaviors vary across the culture, conducting the study in different cultures would make an additional contribution to the literature.

Third, although our study found positive effects of caring behaviors on nurses’ psychological well-being, it is possible to question the effects of caring behavior on work-related outcomes. Therefore, we encourage future studies to extend the benefits of caring behaviors or other-focused behaviors on work engagement, performance, or other work-related outcomes. In the meantime, future research could study mechanisms via which caring behaviors have impacts on well-being or work–related outcomes, such as affective or cognitive pathways.

## Figures and Tables

**Table 1:** Descriptive statistics.

	Mean	SD	1	2	3	4	5	6	7
1. Age	2.38	0.91							
2. Education	1.97	0.80	0.32 **						
3. Position	1.48	0.78	0.60 **	0.28 **					
4. Work hour	40.86	10.02	0.07	0.08	0.13				
5. Bed-use	4.44	1.43	0.00	−0.05	−0.03	0.02			
6. Relational caring	5.06	0.92	0.41 **	0.24 **	0.20 **	0.00	0.03		
7. Vitality	4.89	1.02	0.45 **	0.19 **	0.28 **	0.10	0.06	0.52 **	
8. Moral identification	5.33	0.93	0.38 **	0.26 **	0.21 **	0.08	0.18 **	0.52 **	0.63 **

Note: *n* = 202, ** *p* < 0.01 (two-tailed).

**Table 2:** A result of measurement model test.

Model	χ2	*df*	Δ χ2	χ2/*df*	CFI	TLI	RMSEA	SRMR
One-factor (all items combined)	1363.58	162	1016.79 **	8.41	0.73	0.69	0.19	0.13
Two-factor (moral identification and vitality)	1049.30	161	702.51 **	6.51	0.80	0.76	0.16	0.13
Two-factor (relational caring and vitality)	821.38	161	474.59 **	5.10	0.85	0.82	0.14	0.13
Two-factor (relational caring and moral identification)	697.28	161	350.49 **	4.33	0.88	0.86	0.12	0.13
Three-factor	346.79	159			0.95	0.95	0.07	0.05
Decision criteria			** p < 0.001		>0.95	>0.95	<0.08	<0.08

**Table 3:** The result of regression analysis.

Variable	DV: Vitality (Step 1)			DV: Vitality (Step 2)			DV: Vitality (Step 3)		
	β	*SE*	t	β	*SE*	t	β	*SE*	t
*Control variables*									
Age	0.48 **	0.09	5.42	0.20 **	0.07	2.59	0.17 *	0.07	2.28
Education	0.05	0.08	0.65	−0.08	0.07	−1.18	−0.09	0.07	−1.35
Position	0.00	0.10	0.00	0.05	0.08	0.60	0.05	0.08	0.70
Work hour	0.00	0.00	1.07	0.00	0.00	1.11	0.00	0.00	0.66
Bed-use	0.04	0.04	0.92	−0.02	0.03	−0.74	−0.02	0.03	−0.59
*Testing variables*									
IV: Relational caring				0.24 **	0.06	3.56	0.25 **	0.06	3.73
MV: Moral identification				0.50 **	0.06	7.28	0.51 **	0.06	7.46
IV × MV							0.15 *	0.06	2.48
F			11.06 **			27.38 **			25.37 **
R^2^			0.22			0.49			0.51
Adjusted R^2^			0.20			0.47			0.49
ΔR^2^						0.27 **			0.02 **

Note: *n* = 202; * *p* < 0.05. ** *p* < 0.01; IV= Independent variable, DV = Dependent variable, MV = Moderating variable.

**Table 4:** The moderation effect of moral identification under the condition of 40 and below work hours.

	DV: Vitality (Step 1)			DV: Vitality (Step 2)			DV: Vitality (Step 3)		
Variable	β	*SE*	t	β	*SE*	t	β	*SE*	t
*Control variables*									
Age	0.49 **	0.10	4.61	0.28 **	0.08	3.24	0.25 **	0.08	2.95
Education	0.05	0.98	0.56	−0.04	0.07	−0.53	−0.06	0.07	−0.81
Position	0.00	0.11	0.03	0.00	0.09	0.06	0.01	0.09	0.20
Work hour	0.01	0.00	0.16	0.00	0.00	0.29	0.00	0.00	0.00
Bed-use	0.07	0.05	1.36	0.01	0.04	0.34	0.03	0.04	0.79
*Testing variables*									
IV: Relational caring				0.19 *	0.07	2.51	0.18 *	0.07	2.47
MV: Moral identification				0.51 **	0.07	6.81	0.50 **	0.07	6.91
IV × MV							0.21**	0.06	3.18
F			7.48 **			19.52 **			19.53 **
R^2^			0.21			0.50			0.54
Adjusted R^2^			0.18			0.48			0.51
ΔR^2^						0.30 **			0.03 **

Note: *n* = 141; * *p* < 0.05. ** *p* < 0.01; IV = Independent variable, DV = Dependent variable, MV = Moderating variable.

**Table 5:** The moderation effect of moral identification under the condition of 40+ work hours.

	DV: Vitality (Step 1)			DV: Vitality (Step 2)			DV: Vitality (Step 3)		
Variable	β	*SE*	t	β	*SE*	t	β	*SE*	t
*Control variables*									
Age	0.51 **	0.17	2.88	0.00	0.17	0.00	−0.02	0.17	−0.12
Education	0.10	0.17	0.56	−0.20	0.15	−1.30	−0.20	0.15	−1.33
Position	−0.04	0.22	−0.20	0.19	0.18	1.04	0.20	0.19	1.07
Work hour	0.01	0.02	0.66	0.01	0.01	0.90	0.01	0.01	0.78
Bed-use	−0.02	0.09	−0.24	−0.09	0.08	−1.12	−0.10	0.08	−1.19
*Testing variables*									
IV: Relational caring				0.43 *	0.16	2.64	0.44 *	0.16	2.68
MV: Moral identification				0.50 **	0.16	3.10	0.52 **	0.16	3.12
IV × MV							0.07	0.14	0.54
F			3.40 **			8.33 **			7.23 **
R^2^			0.23			0.52			0.52
Adjusted R^2^			0.16			0.46			0.45
ΔR^2^						0.30 **			−0.74

Note: *n* = 61; * *p* < 0.05. ** *p* < 0.01; IV = Independent variable, DV = Dependent variable, MV = Moderating variable.

## Data Availability

Data is available from the corresponding author by reasonable request.
